# OnabotulinumtoxinA: An Effective Tool in the Therapeutic Arsenal for Chronic Migraine With Medication Overuse

**DOI:** 10.3389/fneur.2018.00808

**Published:** 2018-10-16

**Authors:** Edoardo Caronna, Victor José Gallardo, Natalia Hernández-Beltrán, Marta Torres-Ferrus, Patricia Pozo-Rosich

**Affiliations:** ^1^Headache Unit, Neurology Department, Hospital Universitari Vall d'Hebron, Barcelona, Spain; ^2^Headache and Neurological Pain Research Group, Vall d'Hebron Research Institute, Department of Medicine, Universitat Autònoma de Barcelona, Barcelona, Spain; ^3^Neuroclinica and Promedan, Medellín, Colombia

**Keywords:** headache, migraine, medication overuse headache, chronic migraine, OnabotulinumtoxinA

## Abstract

**Objective:** To evaluate the early response of onabotulinumtoxinA as a treatment tool in patients with chronic migraine (CM) and medication overuse (MO).

**Patients and Methods:** This is a retrospective study in patients with CM and MO who received two cycles of onabotulinumtoxinA infiltrations following PREEMPT protocol. We evaluated the efficacy of onabotulinumtoxinA in MO resolution, defined as less than 10 days/month of acute medication intake (triptans, opioids, and combinations) or 15 days/month (non-steroidal anti-inflammatory drugs - and simple analgesics). In addition, we analyzed changes in headache frequency, pain intensity, and headache-related disability (MIDAS scale). A multivariate analysis was carried out to identify factors independently related to MO resolution.

**Results:** We included 139 consecutive patients with CM and MO. After 2 cycles of onabotulinumtoxinA, 73.4% had ≥50% reduction in acute medication intake and 57.6% achieved MO resolution. 7.9% of patients did not use any acute medication after treatment. Even though both MO-ongoing group and MO-resolution group improve in headache frequency, the reduction was significantly higher for the group which discontinued the use of acute medication after onabotulinumtoxinA treatment (*p* < *0.001*). In this group, 73.0% reduced headache frequency ≥50%. Daily headache changed from 71.2 to 23.2% (*p* < *0.001*). Both groups showed an improvement in pain intensity and in MIDAS score (*p* < *0.05*). In the multivariate analysis we observed that MO resolution had an inverse association with medication intake at baseline (OR:0.294, *p* < *0.05*) and a direct association with frequency (OR:20.455, *p* < *0.001*) and MIDAS score (OR: 6.465, *p* < *0.05*) improvements.

**Conclusion:** OnabotulinumtoxinA has an early beneficial effect on the discontinuation of acute medication in a substantial proportion of patients with CM and MO. Therefore, onabotulinumtoxinA might be considered a therapeutic tool in CM with MO.

## Introduction

Migraine is a prevalent medical condition and is the third cause of disability worldwide between 20 and 55 years of age (1, 2). Chronic migraine (CM) affects around the 2% of the population and up to 50% of patients associate acute pain medication overuse (MO) (3, 4). Women are most affected with peak prevalence at age 40 and it has also been observed that this high incidence is inversely proportional to socioeconomic position (5, 6). Hence, the great impact of MO in patients with headache remains a matter of concern since it is associated to poorer response to preventive treatment (7, 8) and worsening in quality of life and higher disability (9, 10).

Nowadays there are several therapeutic approaches to CM associated to MO regarding the (1) type of withdrawal (gradual vs. abrupt), (2) management of the patient (outpatient vs. inpatient), (3) use of preventive treatment (immediate vs. delayed) and (4) presence or absence of a non-pharmacological concomitant treatment (group therapies, behavioral therapy and psychotherapy) (11, 12). Current guidelines for the treatment of CM include the use of onabotulinumtoxinA. As the majority of patients with MO suffer CM, it might be expected that onabotulinumtoxinA could be a useful treatment in acute medication discontinuation. Although, the use of onabotulinumtoxinA for MO was not specifically evaluated in the PREEMPT clinical trials development (13) nor it is mentioned in Spanish guidelines (14).

After observing in our daily practice that patients seemed to improve their medication overuse after treatment with onabotulinumtoxinA and considering that there are no established therapeutic protocols for these patients, we decided to analyze the efficacy of two cycles of onabotulinumtoxinA as a treatment in patients with CM and MO.

## Methods

This is a retrospective study done in a specialized Headache Clinic in a daily practice setting. We included consecutive outpatient subjects who were attended between July 2014 and December 2017 with the diagnosis of CM with MO who had received treatment with onabotulinumtoxinA as part of their preventive treatment. CM was defined according to the International Classification of Headache Disorders, 3rd edition beta version criteria (ICHD-IIIβ) (15). We defined MO as acute medication intake of ≥10 days/month for triptans, combinations or opioids and of ≥15 days/month for non-steroidal anti-inflammatory drugs (NSAIDs) or simple analgesics (e.g., paracetamol) for at least 3 months. Frequency and number of pills taken per month were specified for each analgesic group.

Data from the initial visit, such as demographic and headache characteristics, was collected. We included patients with concomitant oral preventive treatment maintained on stable dose during the two-cycle set of injections. We did not exclude patients who had received additional treatment with fixed-dose corticosteroids in a 12-day taper down cycle in addition to the first application of onabotulinumtoxinA, if they had previously failed on stopping medication overuse. No other discontinuation therapies were done in the patients included in the study, however in our outpatient clinic visits patients were briefly told as a part of the clinical practice to try to stop medication overuse. Patients were excluded if they had previously received onabotulinumtoxinA for any reason.

Two cycles of onabotulinumtoxinA were administered following the PREEMPT protocol (baseline and after 12 weeks) at a fixed-dose of 155 U and in fixed-sites. The response to onabotulinumtoxinA was evaluated after 6 months from baseline visit and the following data was collected: headache frequency, migraine days/month (MDM), headache days/month (HDM). A migraine day was defined by the patient as any day with intense headache or/and headache with migraine features as photophobia, phonophobia, nausea, vomiting or worsering with physical activity. A headache day was defined as any headache lasting at least 30 min and not defined as a migraine day by the patient. Headache frequency was considered as the sum between headache and migraine days per month. We also collected data in regards to pain intensity, medication intake and Migraine Disability Assessement (MIDAS) score. MO resolution after treatment with onabotulinumtoxinA was defined as an intake <10 days/month for triptans, analgesics combinations or opioids or as an intake <15 day/month for NSAIDs or simple analgesics. Finally, patients also rated headache intensity improvement in 4 categories (No improvement, <25%, 25–49%, ≥50% intensity reduction) after treatment. The improvement in the other variables analyzed was also divided into 4 categories (No improvement, <25%, 25–49%, ≥50%) from the data observed, referring to “no improvement” as the absence of changes in the values between baseline and after treatment.

### Statistical analysis

Descriptive and frequency statistical analysis were obtained and comparisons were made by use of the SPSS statistical package, 23.0 version.

Statistical significance for intergroup differences was assessed using Pearson's chi-square test or Fisher's exact test for categorical variables, the linear trend chi-square test for ordinal variables and the Student's t test or Mann-Whitney U test for continuous variables. Variables associated in the bivariate analysis were entered into an adjusted multivariate logistic regression model to identify factors independently associated with the resolution of MO. A *p* < 0.05 was considered statistically significant.

## Results

Chart review of the prespecified period identified a total of 139 patients treated with onabotulinumtoxinA who fulfilled ICHD-IIIβ CM criteria and MO definition. The mean age was 47.3 ± 11.4 years (18–76) and 82.7% were female. The average time of migraine disease was 30.0 ± 14.1 years (2–62) and the average time of chronification was 9.3 ± 9.0 years (0–52). The main comorbidities observed in our cohort were anxiety (69.1%) and depression (44.9%) (see Table 1).

**Table 1:** Demographic characteristics.

	**n = 139**
Female, n (%)	115 (82.7%)
Age, (Mean ± SD)	47.3 ± 11.4
Age onset, (Mean ± SD)	17.3 ± 11.1
Aura, n (%)	38 (27.3%)
Depression, n (%)	62 (44.9%)
Anxiety, n (%)	96 (69.1%)
Fibromyalgia, n (%)	24 (17.4%)
Chronic fatigue syndrome, n (%)	14 (10.1%)
Sleeping disorders, n (%)	44 (32.4%)

In our cohort, 71.2% of patients had daily headache and 66.2% were using acute medication daily. Seventy-nine point nine percent had previously tried at least one preventive treatment (topiramate and/or beta blocker). Seventy-four percent were taking a preventive treatment at the moment of the first set of injections, being neuromodulators (topiramate, zonisamide, valproic acid, pregabalin) mainly used (57.0%), followed by beta blockers (40.2%) and amitriptyline (28.3%). Twenty-seven patients (19.4%) received corticosteroids together with first dose of onabotulinumtoxinA (see Table 2).

**Table 2:** Baseline migraine characteristics.

**HEADACHE FREQUENCY**	
**HDM**, (Mean ± SD)	13.8 ± 9.0
**MDM**, (Mean ± SD)	13.4 ± 8.1
**Headache frequency**, (Mean ± SD)	27.3 ± 4.7
**Headache frequency**, n (%)16–20 days/month	23 (16.6%)
21–29 days/month	17 (12.2%)
Daily	99 (71.2%)
**ACUTE MEDICATION INTAKE**	
**Corticosteroids use**, n (%)	27 (19.4%)
**Analgesic use**, (Mean ± SD), tablets/month	63.4 ± 58.1
**Analgesic frequency**, n (%) 11–15 days/month	4 (2.9%)
16–20 days/month	25 (18.0%)
21–29 days/month	18 (12.9%)
Daily	92 (66.2%)
**Analgesic classes**, n (%)	
NSAIDs	79 (57.2%)
Triptans	73 (52.8%)
Simple analgesics	52 (37.7%)
Ergots	5 (3.8%)
**PREVENTIVE TREATMENT**	
**Concomitant preventive treatment**, n (%) No treatment	36 (26.0%)
1 treatment	21 (15.1%)
2 treatments	29 (20.8%)
>2 treatments	53 (38.1%)
**DISABILITY**	
**MIDAS**, (Mean ± SD)	80.4 ± 67.5

*MDM, Migraine days/month; HDM, Headache days/month; NSAIDs, non-steroidal anti-inflammatory drugs; MIDAS, Migraine Disability Assessement score*.

After two sets of onabotulinumtoxinA injections 80 patients (57.6%) no longer satisfied MO definition (MO-resolution group) while 59 patients (42.4%) kept overusing acute medication (MO-ongoing group). We did not find any relevant difference on baseline characteristics between groups (MO-resolution *vs*. MO-ongoing), except for fibromyalgia (11.3 vs. 25.4% *p* < *0.05*) and chronic fatigue syndrome (5.0 vs. 16.9% *p* < *0.05*) which were more frequent in the MO-ongoing group.

**Figure 1:**
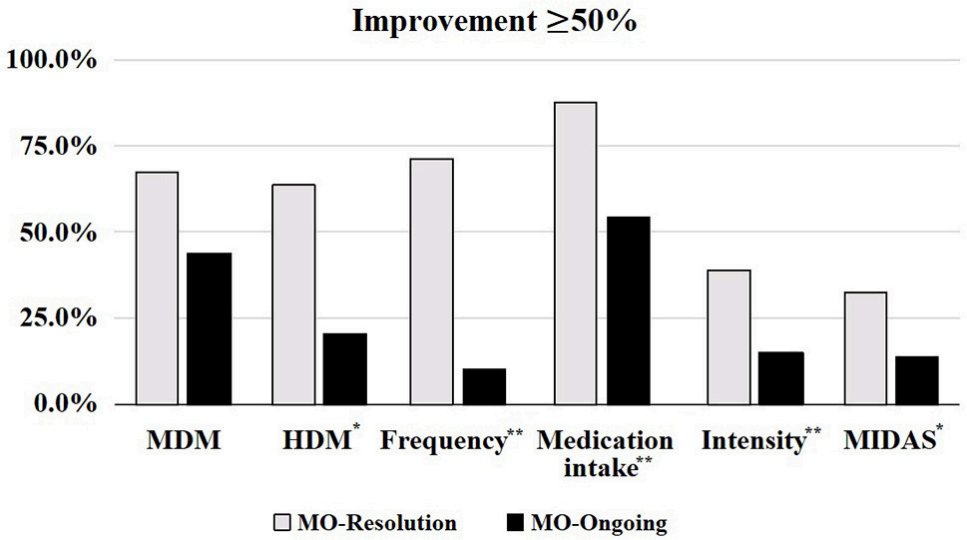
Fifty percent positive response to onabotulinumtoxinA in regards to MO resolution. MDM, Migraine days/month; HDM, Headache days/month; Frequency, headache frequency; MIDAS, Migraine Disability Assessement score; MO, medication overuse. *p < 0.05, **p < 0.001.

The variables analyzed to evaluate the response to onabotulinumtoxinA are (Figure 1):

### Headache frequency

After two sets of onabotulinumtoxinA injections, 63 patients (45.3%) reduced headache frequency ≥50% (*p* < *0.001*). The mean headache frequency decreased from 27.3 ± 4.7 to 15.4 ± 9.9 days/month and patients with daily headache went from 71.2% to 23.2% (*p* < *0.001*). The mean MDM frequency decreased from 13.4 ± 8.1 to 6.5 ± 5.7 days/month, while HDM from 13.8 ± 9.0 to 8.9 ± 8.1 days/month (*p* < *0.001*).

Comparing patients with MO-resolution and MO-ongoing, both groups reduce headache frequency compared to baseline but a ≥50% reduction in headache frequency was significantly higher in the group which discontinued the use of acute medication after onabotulinumtoxinA (MO-resolution 73.0 vs. MO-ongoing 15.3%; *p* < *0.001*). Regarding MDM, both groups reduced significantly the frequency compared to pre-treatment values, but no statistical difference was observed between the two groups. However, when analyzing the HDM, the MO-resolution group showed a significant higher mean reduction in comparison to the MO-ongoing one (65.4 vs. 18.0%; *p* < *0.001*).

**Figure 2:**
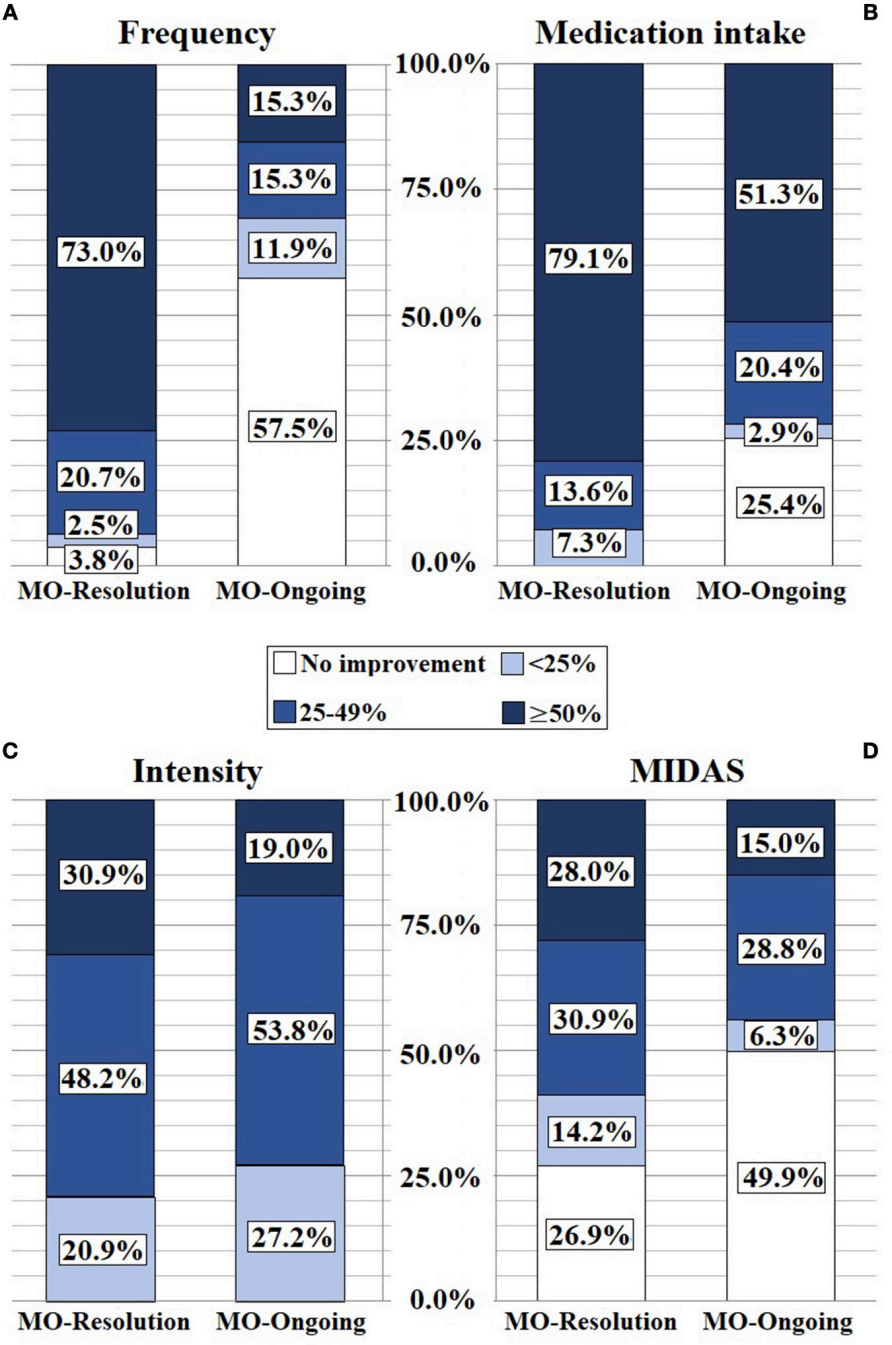
OnabotulinumtoxinA improvement in frequency **(A)**, medication intake **(B)**, intensity **(C)**, and MIDAS **(D)**. MIDAS, Migraine Disability Assessement score; MO, medication overuse.

Despite the fact that they were not overusing acute medication anymore, 6 patients within the MO-resolution group continued suffering from more than 20 days of headache per month. We did a subanalysis in this group of patients but due to its small number we didn't observe any significant result (Figure 2A).

### Acute medication intake

After treatment with onabotulinumtoxinA, 73.4% of the population in our cohort reduced acute medication intake ≥50%: 57.6% (80 patients) no longer fulfilled MO definition. We observed a mean reduction in analgesic intake from baseline of 64.5%. Daily analgesic intake decreased from 66.2 to 13.7% (*p* < *0.001*) and we observed 7.9% of patients who did not take any acute medication.

Both subgroups significantly reduced the analgesic use, yet a ≥50% reduction in acute medication intake was significantly higher in the MO-resolution group in comparison to the MO-ongoing one (79.1 vs. 51.3%; *p* < *0.001*). However, despite keeping to overuse medication, 51.3% of patients in the MO-ongoing group had a ≥50% reduction in analgesic intake (Figure 2B).

In order to evaluate a change in response to acute treatment when used in a non-overuse frequency pattern, we compared the mean number of pills taken per MDM before and after onabotulinumtoxinA in both subgroups, observing a significative reduction in the number of pills taken per MDM only in the MO-resolution group (MO-resolution group 2.1 ± 1.7 vs. 1.2 ± 1.0, *p* < *0.001*).

A multivariate analysis was carried out to identify factors independently related to MO resolution, observing an inverse association with analgesic intake at baseline (OR: 0.294 [0.139-0.625], *p* < *0.05*) and a direct association with frequency (OR: 20.455 [5.913, 70.757], *p* < *0.001*) and MIDAS score (OR: 6.465 [1.366, 30.600], *p* < *0.05*) improvements.

### Headache intensity

Thirty-six point four percent of patients had a ≥50% intensity improvement. Despite the ≥50% intensity reduction was significantly higher in the MO-resolution group in comparison to the MO-ongoing one (30.9 vs. 19.0% *p* < *0.05*), no significant difference was found when analyzing for >25% intensity reduction (Figure 2C).

### MIDAS score

There was a significant improvement compared to pre-treatment in our cohort (80.4 ± 67.5 to 44.4 ± 40.7; *p* < *0.001*). Comparing the two subgroups, a ≥50% improvement in the MIDAS score was significantly higher in the MO-resolution group (28.0 vs. 15.0%; *p* < *0.05*) (Figure 2D).

Considering the possible concomitant effect of corticosteroids in the efficacy variables, we did a subgroup analysis comparing patients who had received corticosteroids and onabotulinumtoxinA with those who had only received onabotulinumtoxinA. No difference was observed between the two groups, except for analgesic use at baseline, showing a daily intake in 96.3% of patients who received corticosteroids compared to 60.0% in those only treated with onabotulinumtoxinA (*p* < *0.001*).

For the same reason, we did another subgroup analysis comparing patients who were taking oral preventive treatment with those without prophylactic drugs and no difference was found in any of the efficacy variables analyzed.

## Discussion

Acute medication overuse is frequently associated with CM with no clearly demonstrated specific treatment. However, amongst some of the possible treatment strategies, onabotulinumtoxinA has been considered since the PREEMPT subgroup analysis of patients with MO showed its effectiveness compared to placebo (16). Further studies have reported similar efficacy of onabotulinumtoxinA in patients with or without MO (17, 18). In our study, baseline demographics and headache characteristics of our cohort were similar to previous published studies (16). We decided to include patients who were taking other preventive therapies at baseline and in whom onabotulinumtoxinA was an add-on treatment, as this is a real-life clinical cohort.

These are our findings:

First, data regarding headache frequency reduction after onabotulinumtoxinA in our cohort confirms that it is an effective treatment for patients suffering from CM and MO.

Our study showed slightly better results in the ≥50% reduction in MDM frequency compared to the patients treated in the PREEMPT subgroup (16) (59.3 vs. 47.2%). On the contrary, similar results were observed in the ≥50% reduction in headache frequency (45.3 vs. 45.8%). Other recent studies (18, 19) have shown an efficacy similar to PREEMPT.

Second, onabotulinumtoxinA clearly reduces medication overuse and analgesic intake in CM patients with MO, which demonstrates the therapeutic efficacy for analgesic discontinuation.

We observed that after onabotulinumtoxinA, 57.6% of patients no longer satisfied MO definition. This result shows higher response to onabotulinumtoxinA compared to the PREEMPT subgroup analysis in patients with MO (16) in which at 6-month post-treatment the percentage of patients with a sustained shift from acute medication overuse to no acute medication overuse represented a 43.4%. However, this is similar to what Aicua et al. (19) observed in a clinical cohort, showing 61.9% of patients who discontinued MO at 6 months.

In our study, a mean reduction in analgesic intake from baseline of 64.5% was observed at 6 months. Guerzoni et al. (20) detected a 20.6% reduction at 6 months, but a further improvement, reaching 67.0%, was achieved at 18 months and not at first injections. Other studies also demonstrated a long-term improvement with repeated cycles of onabotulinumtoxinA (18, 20, 21).

Even if we allowed patients on a stable preventive medication to continue with treatment, the better results observed, especially in MO resolution, cannot be explained by this since no differences were found in our subgroup analysis comparing patients with and without prophylactic treatment. Similarly, administering a short corticosteroid cycle didn't correlate with a better outcome. Current preventive treatment was also maintained in other studies (18), but no comparative analysis was done between patients on concomitant oral preventive treatment and those without it. Contrary to our results, the COMPEL study was the only one to demonstrate (22) that patients on oral preventive treatment at baseline had slightly smaller reduction in headache frequency after onabotulinumtoxinA compared to those without prophylactic drugs, maybe due to the fact of suffering from more severe CM.

Finally, onabotulinumtoxinA reduces frequency, intensity and disability even when medication overuse is ongoing.

As part of our analysis we decided to compare patients who had discontinued acute medication overuse and those still overusing it after treatment with onabotulinumtoxinA. There are no studies which have compared these two subgroups. We demonstrated that a remarkable percentage of patients achieve a ≥50% reduction in headache frequency, both in MDM and HDM, as well as in headache intensity, medication intake frequency and MIDAS score in both groups. This result supports the effectiveness of onabotulinumtoxinA in CM with MO, even in those patients who did not stop acute medication overuse. Even if our study demonstrates that the group who discontinued medication overuse achieves statistically greater improvement compared to the MO-ongoing group in all the efficacy variables analyzed, it is important to continue offering treatment with onabotulinumtoxinA to patients who continue to overuse medication as they also benefit from it.

Furthermore, a multivariate analysis was done to identify factors associated to MO discontinuation, showing that the lower analgesic intake at baseline the easier it was to achive MO resolution. No difference in baseline characteristics was found between the patients that discontinued acute medication overuse and those who did not, not being this a predictor of response to onabotulinumtoxinA. In regards to treatment prediction, a Spanish multicenter study including patients with CM treated with onabotulinumtoxinA observed that unilaterality of pain, fewer days of disability at onset, milder headache at baseline and a <12 months since beginning of chronification were correlated to a better outcome (23); however no specific subgroup analysis was performed in the MO subgroup of patients.

Hence, our study supports the importance of achieving analgesic medication discontinuation through onabotulinumtoxinA, as patients who discontinue have better outcomes. It has to be emphasized as well, that patients who stopped overusing medication with the help of onabotulinumtoxinA noticed a greater response to acute treatment when used in a non-overuse frequency pattern.

A limitation of our study is the absence of a control arm which makes it difficult to establish whether the discontinuation of medication is a direct effect of onabotulinumtoxinA on the reduction in headache days or a contributing cause, together with onabotulinumtoxinA, to the improvement of headache. In other words, it may be complicated to assess if certain patients have a higher response in efficacy to onabotulinumtoxinA, which could alone lead to a more satisfactory discontinuation, or if they combine the effect of onabotulinumtoxinA and self-controlled medication intake, considering as well the uncertain impact on patients of a brief advice concerning stopping medication overuse. However, it is certain that onabotulinumtoxinA alone is effective in achieving detoxification, as it was already demonstrated in the PREEMPT subgroup analysis were no education was done (16). Our study reflects this in a real-life clinical setting. There is a similar study to ours (24), which analyzes the use of onabotulinumtoxinA associated to an acute medication intake. However, the 68 patients with MO and CM were randomized to onabotulinumtoxinA or placebo, after undergoing analgesic medication detoxification, something which is in essence different. The study showed a significant reduction in the number of days of analgesic consumption in the group who were injected with onabotulinumtoxinA, leading to the conclusion that the efficacy of early discontinuation plus onabotulinumtoxinA is superior to early discontinuation alone in reducing acute medication intake.

In a real-life clinical cohort, onabotulinumtoxinA is efficacious and a suitable option in the therapeutic arsenal of CM associated to MO treatment. However, further randomized controlled clinical trials comparing strategies should be done to properly assess the specific effect of onabotulinumtoxinA in this cohort of patients.

## Conclusions

OnabotulinumtoxinA is an effective treatment in CM associated to MO. Our data from a real-life clinical practice setting confirms the efficacy and safety of previous studies. This study also highlights the usefulness of onabotulinumtoxinA as a tool in the therapeutic arsenal for acute medication discontinuation.

## Ethics statement

The study was approved by the Vall d'Hebron Ethics Committee (PR(AG)05/2017). All patients consented to receive treatment with onabotulinumtoxinA and, at the same time gave a written informed consent for further analysis of patients' data which was collected according to Spanish regulation on clinical trials.

## Consent to publish

All patients consented to publication of anonymous individual data.

## Author contributions

PP-R and MT-F made substantial contributions to conception and study design. NH-B, MT-F and PP-R worked for acquisition of data. VJG contributed to analysis and interpretation of data. EC, VG, and NH-B wrote first draft. PP-R and MT-F critically revised and finally approved the version to be published. All authors fully comply with and approve the version to be published.

### Conflict of interest statement

PP-R has received honoraria as a consultant and speaker for: Allergan, Almirall, Chiesi, Eli Lilly, Janssen Cilag, MSD, Novartis and Teva. Her research group has received research grants from Allergan and has received funding for clinical trials from Alder, Boeringher Ingelheim, MSD, Electrocore, Eli Lilly, Janssen Cilag, Novartis. She is a trustee member of the board of the International Headache Society, she is the Coordinator of the Spanish Headache Study Group of the Spanish Neurological Society. She is in the editorial board of Revista de Neurologia. She is an editor for Frontiers of Neurology and Journal of Headache and Pain. She is a member of the Clinical Trials Guidelines Committee of the International Headache Society. She has edited the Guidelines for the Diagnosis and Treatment of Headache of the Spanish Neurological Society. She is the founder of www.midolordecabeza.org. PP-R does not own stocks from any pharmaceutical company. MT-F has received honoraria from Allergan plc, Novartis, Chiesi. The remaining authors declare that the research was conducted in the absence of any commercial or financial relationships that could be construed as a potential conflict of interest.
